# Chemical Defense in Marine Organisms

**DOI:** 10.3390/md18100518

**Published:** 2020-10-18

**Authors:** Chiara Lauritano, Adrianna Ianora

**Affiliations:** Department of Marine Biotechnology, Stazione Zoologica Anton Dohrn, CAP80121 Naples, Italy; ianora@szn.it

## 1. Introduction

Marine organisms are constantly exposed to variations in physical parameters (e.g., temperature and salinity), chemical communication metabolites and/or environmental contaminants and have evolved several mechanisms to survive in extremely different environments. Stressors may alter cellular homeostasis, causing oxidative stress, reproductive failure, disorientation and, in worst cases, organism death. Cells respond to these stimuli by activating a series of defense strategies in order to restore cellular homeostasis and avoid negative impacts [1]. The adopted defense strategies may range from the activation of defensive proteins, such as heat shock proteins (HSPs) and adenosine triphosphate (ATP)-binding cassette (ABC) proteins, as well as the activation of detoxification enzymes and other metabolic pathways responsible for the synthesis of defense compounds/toxins (e.g., antipredator compounds).

HSPs are highly conserved proteins activated in response to various environmental stress factors [2,3,4]. For example, HSP70 can be involved in the tolerance of hyperthermia, ischemia/hypoxia, resistance to hydrogen peroxide, escape from drug-induced cell cycle arrest, tolerance to ultraviolet radiation and apoptosis [3,4]. ABC proteins are a family of energy-dependent efflux protein pumps which act as efflux pumps, leading to lower intracellular accumulation of xenobiotic substrates [5]. ABC proteins have been detected in several marine organisms, including sponges, mussels, oysters, crabs, worms, sea stars, clams and fishes [1,6,7,8], exposed to different environmental stressors (e.g., exposure to copper for the reef coral *Montastraea franksi* [9] and to hydrocarbons, pesticides and heavy metals for the fish *Mugilogobius abei* exposed to [10].

Stress exposure is known to induce an increase in free radicals, such as reactive oxygen species (ROS), which may induce damage to DNA, RNA, proteins, lipids and carbohydrates. Various enzymes are involved in ROS detoxification, such as superoxide dismutase (SOD) and catalase (CAT), which have been found activated upon stress exposure in various marine organisms [1,11,12,13,14]. Glutathione, synthetized by glutathione synthetase, is an important cell scavenger compound involved in radical compound deactivation, and can be found in reduced and oxidized states [15]. The oxidized state can be converted, again, into the reduced state due to the enzyme glutathione reductase (GR) that renders the thiol group of cysteinyl residue available as a source of one reducing equivalent [16]. Glutathione S-transferase (GST) enzymes catalyze glutathione conjugation to lipophilic molecules which need detoxification.

In addition to these defense molecules and enzymes, marine organisms are known to produce a great variety of compounds, unique in terms of diversity, structural and functional features, which have been shown to have defensive roles (the best known of which are marine toxins) [17,18,19]. In addition, several of these compounds have been tested for different bioactivities (e.g., anticancer, anti-inflammatory and antioxidant, as well as for the treatment of neurodegenerative diseases) and results have shown their potential for possible industrial applications [20,21,22,23,24,25,26,27,28,29,30,31,32]. Several experiments and observations have focused on understanding defense strategies of marine organisms and the identification of new natural products by using various physiological, chemical, as well as -omics approaches [33,34,35,36]. This Special Issue aims to highlight recent discoveries on defensive strategies adopted by marine organisms, from micro- to macroorganisms.

## 2. Special Issue Findings

The issue includes two studies on the effects of diatoms, a major group of microalgae found in the world’s oceans, on their predators. Previous studies had already shown that several species of diatoms produce secondary metabolites, derived from the oxidation of fatty acids known as oxylipins, which induce reproductive impairment and teratogenic effects on their predators [4,37,38,39,40,41]. In this Special Issue, Asai et al. [42] report, for the first time, the de novo assembled transcriptome of the calanoid copepod *Calanus helgolandicus* feeding on the oxylipin-producing diatom *Skeletonema marinoi*. Differential expression analysis was performed between copepods exposed to the diatom and the control flagellate *Prorocentrum minimum*, which does not produce oxylipins. Results showed that transcripts involved in carbohydrate, amino acid, folate and methionine metabolism, embryogenesis and response to stimulus were differentially expressed. Data were confirmed on 27 selected genes belonging to these functional categories in *C. helgolandicus* exposed to a mixed solution of pure oxylipins, heptadienal and octadienal, at concentrations of 10, 15 and 20 µM. Results confirmed the up-regulation of genes involved in the stress response and down-regulation of genes associated with folate and methionine metabolism, embryogenesis and signaling, giving new insights on mechanisms of activity of oxylipins.

The issue also reports the findings of Albarano et al. [43], who tested the effects of a mixture of the oxylipins 5-, 9-, 11-, and 15-hydroxyacids (HEPEs), showing that they induced synergistic effects such as increased malformations in sea urchin embryos at lower concentrations and increased delay of embryogenesis. At the gene level, oxylipin mixtures induced expression variations in 50 genes involved in different functional processes.

Another series of papers investigate the effects of toxic dinoflagellates that produce a series of toxins with deleterious impacts on human and environmental health, with devastating effects on local economies. Vingiani et al. sequenced the full transcriptome of the dinoflagellate *Alexandrium tamutum*. The clone was not known to produce saxitoxins but was known to induce copepod reproduction impairment and antiproliferative activity on human cells [22,44]. Results identified the presence of three transcripts related to saxitoxin synthesis (sxtA, sxtG and sxtU) and other transcripts potentially related to the synthesis of additional toxic compounds (e.g., 44 transcripts annotated as “polyketide synthase”), suggesting that this species may produce other toxic metabolites. Considering that this study highlights the presence of metabolic pathways that can produce toxic compounds, and that *Alexandrium tamutum* was previously found active against human cancer cells, these data suggest the need to further investigate this species for the possible discovery of new drug lead compounds.

*Prorocentrum lima* is another toxic dinoflagellate, which can produce phycotoxins such as okadaic acid (OA). Gu and co-workers [45] here identify three ABC transporter genes (ABCB1, ABCC1 and ABCG2) and characterize their expression patterns as well as OA production under different environmental conditions in *P. lima*. For example, they observed that Cu^2+^ exposure could up-regulate ABCB1, ABCC1 and ABCG2 transcripts, suggesting a defensive role of ABC transporters against metal ions in surrounding waters. In addition, they found that Cu^2+^, as well as the presence of the grazer *Artemia salina*, could induce OA production. Although their data provide new molecular insights on the defensive responses of *P. lima* to the surrounding environment, the authors did not find a correlation between OA production and ABC transporter expression patterns.

Bivalves are filter-feeding animals, mainly ingesting microalgae that can accumulate paralytic shellfish toxins (PSTs) produced by harmful algae. In this Special Issue, Lian and co-workers [46] performed the first systematic analysis of SOD genes in the bivalve *Chlamys farreri*, an important aquaculture species in China. A total of six Cu/Zn-SODs (SOD1-6) and two Mn-SODs (SOD7, SOD8) were identified. Expression regulation of SOD genes was analyzed in the hepatopancreas and kidney of scallops exposed to two different PST-producing dinoflagellates, *Alexandrium minutum* and *Alexandrium catenella*. In both tissues, and after exposure with both dinoflagellates, an increase in almost all SODs was observed, suggesting their importance in protecting scallops from the stress induced by PSTs.

Patel et al. [47] studied the mucus of the skin of the Atlantic salmon *Salmo salar* because it plays a vital role in innate immune defense. Some mucus proteins can incapacitate pathogens and/or inhibit their passage through the skin. Patel et al. isolated and characterized galectin(s), β-galactoside-binding proteins, present in the mucus. In particular, a novel short form of galectin-3 was isolated and, considering that mass spectrometric analysis showed that the isolated protein was the C-terminal half of galectin-3, it was named galectin-3C. Galectin-3C was able to agglutinate the Gram-negative pathogenic bacteria *Moritella viscosa*. In fact, *M. viscosa* incubated with galectin-3C modified its proteome, changing the abundance of multidrug transporters (belonging to the ABC protein family) and three ribosomal proteins L7/12, S2, and S13. Overall, their study suggested a possible role of galectin-3 in the immune defense of Atlantic salmon.

Wu and co-workers [48] report a detailed chemical investigation of two nudibranchs, *Phyllidiella pustulosa* and *Phyllidia coelestis*, from the South China Sea, as well as of their possible interactions with their sponge-prey *Acanthella cavernosa*. The study has led to the isolation of various marine natural products. In particular, one new nitrogenous cadinane-type sesquiterpenoid xidaoisocyanate A, one new nitrogen-containing kalihinane-type diterpenoid bisformamidokalihinol A and 17 known nitrogenous terpenoids were identified and their structures elucidated. In addition, bioactivity testing on four human cancer cell lines (i.e., lung A549, colon HT-29, liver SNU-398 and pancreas Capan-1 cell lines) showed cytotoxicity induced by incubation with bisabolane-type sesquiterpenoids 8, 10 and 11. The authors suggest that these metabolites are possible chemical nudibranch defensive molecules used to survive predation pressure in harsh marine environments.

Finally, D’Ambra and Lauritano [49] review toxins produced by Cnidaria, their molecular weights, biological activities and possible biotechnological applications. Their study also shows how recent-omics studies, which have become more common in the last ten years, have allowed the identification of several new toxins from Cnidaria. They showed how a plethora of Cnidaria compounds acting on different targets have suggested potential applications for the treatment of neurodegenerative diseases, epilepsy and acute and chronic pain, as well as cancer.

As editors of this Special Issue, we hope that it will provide a unique and valuable reference source for every researcher interested in this research topic, and we express our sincere gratitude to all authors who contributed to rendering this a very Special Issue with new and exciting discoveries on chemical defenses in marine organisms.
